# *Aronia* Bioactive Fraction-Alginic Acid Nanocomplex-Modulates Tau Phosphorylation and Aggregation in Cell Models of Alzheimer’s Disease

**DOI:** 10.3390/ijms27135748

**Published:** 2026-06-25

**Authors:** Hye-Yeon Kang, Bong-Keun Jang, Seong-Hoon Yun, Hee-Yeong Jeong, Eunkuk Park, Kang-Il Oh, Junhwan Jeong, Seon-Yong Jeong

**Affiliations:** 1 JBKLAB, Inc., 17 Techno 4-ro, Yuseoung-gu, Daejeon 34013, Republic of Korea; khy@cellmed.com (H.-Y.K.); seonghoon.yun@cellmed.com (S.-H.Y.); heeyeong.jeong@cellmed.com (H.-Y.J.); 2 Department of Medical Genetics, Ajou University School of Medicine, Suwon 16499, Republic of Korea; eunkuk0815@daum.net (E.P.); kyl213@ajou.ac.kr (K.-I.O.); enung7014@ajou.ac.kr (J.J.); 3 BK21 R&E Initiative for Advanced Precision Medicine, Department of Biomedical Sciences, Graduate School of Ajou University, Suwon 16499, Republic of Korea; 4 Department of Herbology, College of Korean Medicine, Kyung Hee University, 26 Kyungheedae-ro, Dongdaemun-gu, Seoul 02447, Republic of Korea

**Keywords:** Alzheimer’s disease, *Aronia* bioactive fraction-alginic acid nanocomplex (AANCP), *Aronia melanocarpa*, autophagy, glycogen synthase kinase-3β, Tau aggregation, Tau phosphorylation, tauopathy

## Abstract

Preventing or reversing Tau hyperphosphorylation and aggregation represent critical objectives in the development of effective therapies for Alzheimer’s disease. The present study investigated the potential of a novel *Aronia* bioactive fraction—alginic acid nanocomplex (AANCP)—to simultaneously inhibit pathological features of Alzheimer’s disease. Evaluations of *Aronia* bioactive fraction (ABF) and low-molecular-weight alginic acid (LAA), utilized both individually and as AANCP, were conducted in HEK293-TauP301L and SH-SY5Y-TauP301L cell models of Alzheimer’s disease. Both ABF and LAA reduced the expression of total Tau and Tau phosphorylated at Ser396 in a concentration-dependent manner, with AANCP demonstrating significant synergistic activity of its components. Notably, the optimal AANCP ratio was 1:1 and 1:8 for inhibiting Tau phosphorylation and Tau aggregation, respectively. Mechanistically, AANCP inhibited Tau phosphorylation by upregulating p-Akt (phosphorylated protein kinase B) and p-GSK-3β (phosphorylated glycogen synthase kinase-3 beta), while also enhancing the activity of methylated PP2A, a key Tau phosphatase. Furthermore, AANCP exhibited superior efficacy in inhibiting heparin-induced Tau aggregation compared to the individual components. Analysis of autophagy markers indicated that the nanocomplex enhanced Tau clearance, as shown by increased LC3-II and Beclin-1 levels and reduced p62 levels. These results suggest AANCP as a promising therapeutic candidate that simultaneously reduces Tau phosphorylation and aggregation and facilitates autophagic Tau clearance, offering a potent, synergistic strategy for treating Alzheimer’s disease.

## 1. Introduction

The development of effective therapies for Alzheimer’s disease (AD) has increasingly pivoted towards targeting Tau pathology, a core feature of the progressive neurodegenerative disorder [1]. Although the brains of patients with AD are characterized by both amyloid-β (Aβ) plaques and intracellular neurofibrillary tangles of hyperphosphorylated Tau [2,3], decades of clinical trials predominantly targeting Aβ have yielded inconclusive results [4,5]. This failure to halt cognitive decline has intensified the focus on neurofibrillary tangles, as the anatomical burden of these neurofibrillary tangles correlates much more strongly with neuronal loss and clinical dementia severity than that of Aβ plaques [6,7]. Unlike Aβ, the progression of Tau pathology directly reflects the trajectory of cognitive impairment, establishing a more precise therapeutic target [7]. Abnormal hyperphosphorylation impairs Tau’s microtubule-stabilizing function, leading to Tau detachment and subsequent aggregation into toxic fibrils that inflict direct neuronal injury and promote cell death [8,9]. Consequently, strategies aimed at mitigating Tau hyperphosphorylation and promoting the clearance of pathological Tau species have emerged as a more promising frontier in AD research [10].

The dysregulation of Tau phosphorylation stems from a disrupted equilibrium between kinases and phosphatases [11]. Among such kinases, glycogen synthase kinase-3β (GSK-3β) plays a key role in driving tauopathies, phosphorylating Tau at critical sites such as Ser396 and Ser404 [12,13]. Normally, GSK-3β activity is suppressed by the PI3K (phosphoinositide 3-kinase)-Akt (Protein kinase B) signaling pathway to maintain neuronal health. In AD, however, the PI3K-Akt pathway is impaired, leading to uncontrolled GSK-3β activity and, consequently, accelerated Tau pathology [14,15]. The primary enzyme that counteracts this process, protein phosphatase 2A (PP2A), is responsible for the majority of Tau dephosphorylation in the brain but often exhibits reduced function in AD [16,17]. As the activity of PP2A relies on proper methylation, any impairment in the methylation process directly contributes to the pathological buildup of hyperphosphorylated Tau [18]. Furthermore, aggregated Tau is primarily cleared via the autophagy-lysosome pathway. Deficiencies in autophagic clearance, which can be tracked by observing markers like LC3-II, Beclin-1, and p62, result in the accumulation of toxic Tau forms, suggesting enhanced autophagy as another key therapy for tauopathies [19,20].

To block these pathological processes, various therapeutic methods and modulators targeting Tau have been widely developed. Current approaches mainly focus on either chemical kinase inhibitors (such as GSK-3β inhibitors) to prevent Tau hyperphosphorylation, or microtubule-stabilizing agents to physically block Tau peptide aggregation [12,13]. However, many of these conventional small-molecule modulators face limitations due to cellular toxicity or low bioavailability.

*Aronia melanocarpa* (black chokeberry) is particularly notable for its rich content of anthocyanins (primarily cyanidin derivatives) [21], which are known to penetrate the blood-brain barrier and exert strong neuroprotective effects [22]. However, a key hurdle for the clinical translation of these compounds is their inherent instability under physiological conditions and poor bioavailability due to rapid degradation [23,24]. To overcome this obstacle, our study employed a nanotechnology-based strategy, creating a formulation that combines an *Aronia* bioactive fraction (ABF) with low-molecular-weight alginic acid (LAA). LAA is a biocompatible polysaccharide that can spontaneously self-assemble with ABF, forming a nanocomplex engineered to shield its bioactive cargo and improve therapeutic delivery [25,26]. We hypothesized that the novel *Aronia* bioactive fraction-alginic acid nanocomplex (AANCP) would optimize the delivery of anthocyanins to cellular targets, producing a synergistic effect that reduces Tau phosphorylation, inhibits Tau aggregation, and promotes autophagic Tau clearance more effectively than ABF or LAA alone.

## 2. Results

### 2.1. ABF and LAA Reduce the Levels of Phosphorylated Tau in Cellular Models of AD

**Figure 1:**
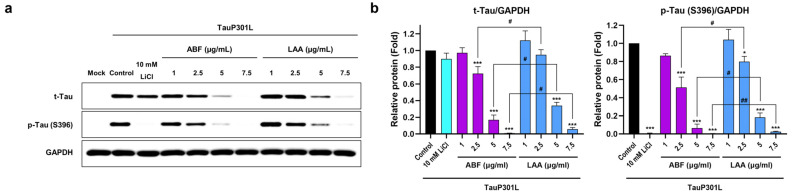
*Aronia* bioactive fraction (ABF) and low-molecular-weight alginic acid (LAA) inhibit Tau phosphorylation in a concentration-dependent manner. (**a**) Representative Western blots showing the effects of various ABF and LAA concentrations (1, 2.5, 5, and 7.5 μg/mL) on the levels of t-Tau and p-Tau (S396) in HEK293-TauP301L cells. (**b**) Western blot analysis and corresponding densitometric quantification showing the concentration-dependent reduction in t-Tau and p-Tau (S396) levels in HEK293-TauP301L cells following 24 h of treatment. Data are expressed as the mean ± SD. Significance was determined by one-way ANOVA (vs. Control). * *p* < 0.05, *** *p* < 0.001 vs. Control. For direct comparisons between ABF and LAA at the same concentration, significance was assessed using an unpaired two-tailed Student’s *t*-test. ^#^ *p* < 0.05, ^##^ *p* < 0.01 vs. LAA. ABF, *Aronia* bioactive fraction; ANOVA, analysis of variance; LAA, low-molecular-weight alginic acid; p-Tau, phosphorylated Tau; SD, standard deviation; t-Tau, total Tau.

To assess the capacity of *Aronia* bioactive fraction (ABF) and low-molecular-weight alginic acid (LAA) to mitigate Tau hyperphosphorylation, total Tau (t-Tau) and the phosphorylated tau variant at Ser396 (p-Tau S396) levels were measured within HEK293-TauP301L cells (Figure 1a). Pathological Tau expression and phosphorylation were induced through transfection with the TauP301L plasmid in the HEK293 cells. Following a 24 h treatment with various concentrations of ABF or LAA, the inhibitory effects of the compounds were evaluated. Treatment with ABF at 2.5, 5.0, and 7.5 µg/mL resulted in a significant decrease in the expression of both t-Tau and p-Tau (S396) compared to the untreated control group. Likewise, LAA showed inhibitory activity, significantly lowering t-Tau expression at LAA concentrations of 5.0 and 7.5 µg/mL, as well as reducing p-Tau (S396) levels at all tested concentrations (2.5, 5.0, and 7.5 µg/mL; Figure 1b). These results indicate that ABF has a more potent inhibitory effect, significantly reducing p-Tau (S396) expression by 35.7% and 65.5% more than LAA at equivalent concentrations of 2.5 and 5.0 µg/mL, respectively. This quantitative difference confirms that ABF functions as the principal active agent within the *Aronia* bioactive fraction-alginic acid nanocomplex (AANCP).

**Figure 2:**
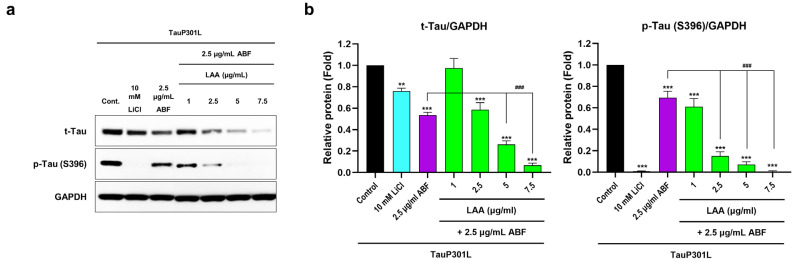
*Aronia* bioactive fraction-alginic acid nanocomplex (AANCP) exhibits a synergistic effect of ABF and LAA in inhibiting Tau phosphorylation. (**a**) Representative Western blot images demonstrating the effects of AANCP on Tau phosphorylation in HEK293-TauP301L cells. (**b**) Western blot analysis and densitometric quantification demonstrating the synergistic reduction in p-Tau (S396) expression in cells treated with AANCP compared with those treated with ABF alone. Data are expressed as the mean ± SD. Significance was determined by one-way ANOVA. ** *p* < 0.01, *** *p* < 0.001 vs. Control; ^###^ *p* < 0.001 vs. ABF alone. AANCP, *Aronia* bioactive fraction-alginic acid nanocomplex.

Building on previous findings that AANCP formulation enhances therapeutic efficacy through improved structural stability [27], we investigated whether the enhanced stability extends to the modulation of Tau phosphorylation. To evaluate the synergistic potential, t-Tau and p-Tau (S396) expression were analyzed in HEK293-TauP301L cells treated with various AANCP formulations (Figure 2a). The nanocomplexes were prepared with a fixed ABF concentration (2.5 μg/mL) and varying amounts of LAA (1.0, 2.5, 5.0, and 7.5 μg/mL). Compared to cells treated with ABF alone, AANCP treatments containing 2.5, 5.0, or 7.5 μg/mL of LAA presented a significantly greater reduction in p-Tau (S396) levels (Figure 2b). This result suggests a synergistic interaction, whereby the combination of ABF and LAA at a 1:1 ratio or higher significantly amplifies the inhibition of Tau phosphorylation beyond the effect of ABF alone.

### 2.2. ABF and LAA Modulate Tau-Related Kinase and Phosphatase Pathways

**Figure 3:**
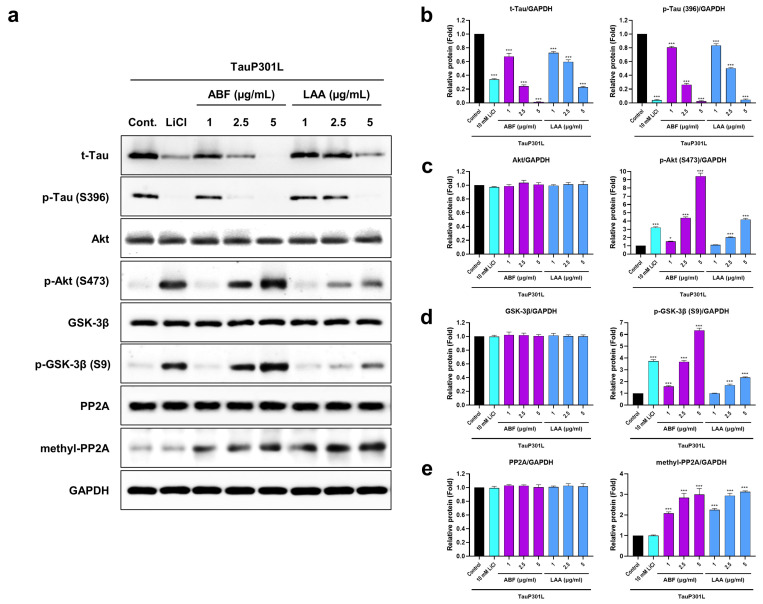
ABF and LAA modulate the Tau regulatory signaling pathway in SH-SY5Y-TauP301L cells. (**a**) Representative Western blot images illustrating the protein levels of key signaling molecules after a 24 h treatment. GAPDH was used as a loading control. (**b**–**e**) Densitometric quantification of the Western blot bands shown in (**a**) for (**b**) p-Tau (S396), (**c**) p-Akt (Ser473), (**d**) p-GSK-3β (Ser9), and (**e**) methylated PP2A. Data are expressed as the mean ± SD. Significance was determined by one-way ANOVA. * *p* < 0.05, *** *p* < 0.001 vs. Control. GSK-3β, glycogen synthase kinase-3β; PP2A, protein phosphatase 2A.

To elucidate the molecular mechanisms underlying the effects of ABF and LAA, we examined key regulators of Tau phosphorylation, including the kinase GSK-3β and the phosphatase PP2A. SH-SY5Y-TauP301L cells were treated with ABF or LAA at three concentrations (1.0, 2.5, and 5.0 μg/mL) for 24 h. Subsequently, the protein expressions of Akt, phosphorylated Akt at Ser473 (p-Akt [Ser473]), GSK-3β, phosphorylated GSK-3β at Ser9 (p-GSK-3β (Ser9)), PP2A, methylated PP2A, t-Tau, and p-Tau (S396) were analyzed (Figure 3a). Both ABF (1.0, 2.5, and 5.0 μg/mL) and LAA (1.0, 2.5, and 5.0 μg/mL) induced a significant decrease in p-Tau (S396) levels in concentration-dependent manners. Concurrently, both ABF and LAA treatments induced a dose-dependent increase in the levels of p-Akt (Ser473) and the downstream target p-GSK-3β (Ser9). Notably, ABF demonstrated a stronger capacity to upregulate p-Akt and p-GSK-3β than LAA, suggesting more potent activity in modulating the Tau-related kinase and phosphatase pathway (Figure 3b–d). Additionally, both compounds prompted a significant, concentration-dependent increase in methyl-PP2A level relative to the control (Figure 3e).

**Figure 4:**
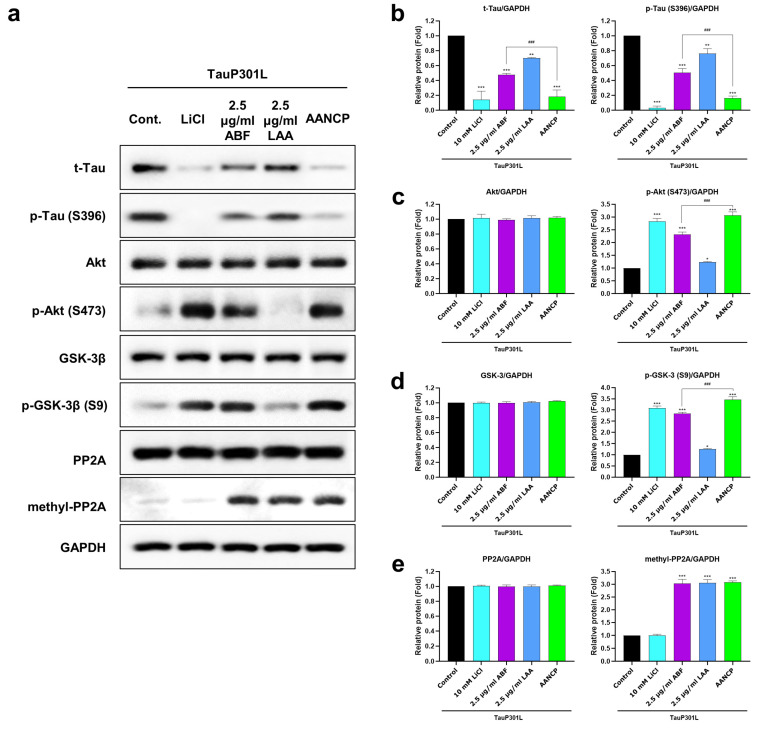
AANCP formulation presents enhanced effects on Tau regulatory signaling compared to individual treatments. (**a**) Representative Western blot images displaying the expression levels of p-Tau (S396), p-Akt (Ser473), p-GSK-3β (Ser9), and methylated PP2A. (**b**–**e**) Corresponding densitometric quantification of the bands for (**b**) p-Tau (S396), (**c**) p-Akt (Ser473), (**d**) p-GSK-3β (Ser9), and (**e**) methylated PP2A. Data are expressed as the mean ± SD. Significance was determined by one-way ANOVA. * *p* < 0.05, ** *p* < 0.01, *** *p* < 0.001 vs. Control; ^###^ *p* < 0.001 vs. ABF alone.

We next evaluated whether AANCP enhances the modulation of the current signaling pathways by comparing nanocomplex activity to that of the individual components, using a 1:1 formulation (Figure 4a). Treatment with AANCP (2.5 μg/mL ABF and 2.5 μg/mL LAA) resulted in a significantly greater reduction in p-Tau (S396) expression compared to treatment with ABF 2.5 μg/mL alone. This superior effect was mirrored upstream, as AANCP also induced significantly higher expression of p-Akt (Ser473) and p-GSK-3β (Ser9) (Figure 4b–d). However, regarding the dephosphorylation pathway, the increased expression of methylated PP2A in the AANCP group was not significantly different from the levels observed in the group treated with ABF alone (Figure 4e).

### 2.3. ABF and LAA Inhibit Heparin-Induced Tau Aggregation

**Figure 5:**
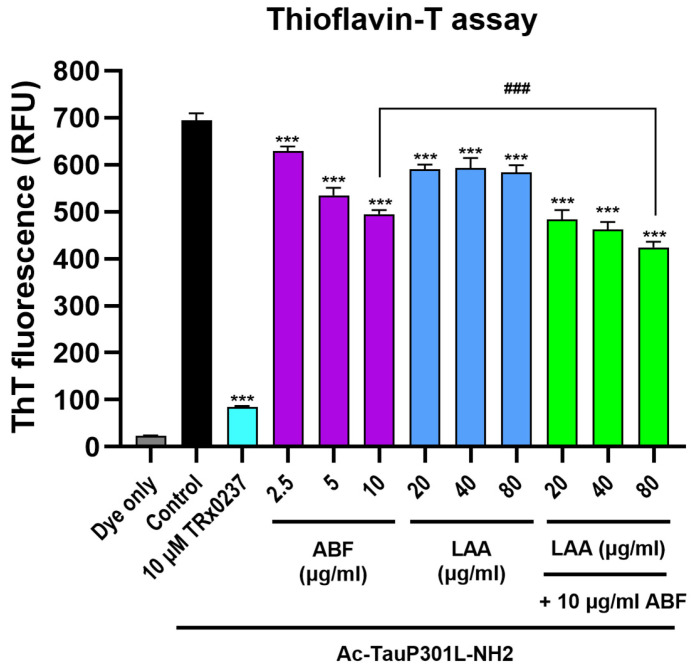
Inhibitory effects of AANCP on Tau aggregation. ThT fluorescence assay results reveal the inhibitory effects of ABF, LAA, and AANCP (1:8 ratio) on heparin-induced TauP301L peptide aggregation. Data are expressed as the mean ± SD. Significance was determined by one-way ANOVA. *** *p* < 0.001 vs. Control; ^###^ *p* < 0.001 vs. ABF alone. RFU, relative fluorescence unit; ThT, Thioflavin T.

Since the Tau hyperphosphorylation reduces protein binding affinity to microtubules and promotes the formation of pathogenic aggregates, we determined whether ABF and LAA inhibit the aggregation process. A thioflavin T (ThT) fluorescence assay was employed to measure the heparin-induced aggregation of TauP301L peptides. ABF significantly inhibited Tau aggregation at all tested concentrations (2.5, 5.0, and 10.0 μg/mL). Likewise, LAA significantly suppressed Tau aggregation at higher concentrations (20, 40, and 80 μg/mL), yet lacked clear concentration-response dependency. Importantly, AANCP formulated at a 1:8 ratio (10 μg/mL ABF and 80 μg/mL LAA) significantly enhanced aggregation prevention compared to the activity of 10 μg/mL ABF alone (Figure 5). This result suggests that the AANCP formulation improves the capacity of ABF to block Tau aggregation.

### 2.4. AANCP Enhances Autophagic Activity

**Figure 6:**
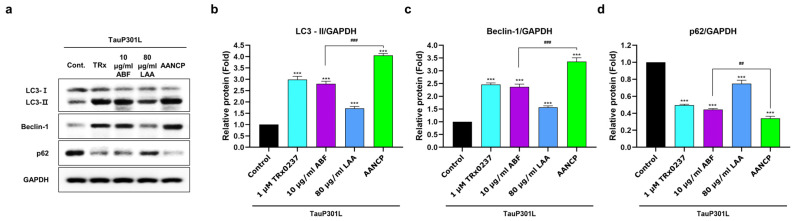
AANCP promotes autophagic processes. (**a**) Representative Western blot images showing the expression levels of autophagy-related markers LC3-I/II, Beclin-1, and p62 after 24 h of treatment. GAPDH was used as a loading control. (**b**–**d**) Densitometric quantification of (**b**) LC3-II, (**c**) Beclin-1, and (**d**) p62 protein expression levels. Data are expressed as the mean ± SD. Significance was determined by one-way ANOVA. *** *p* < 0.001 vs. Control; ^##^ *p* < 0.01, ^###^ *p* < 0.001 vs. ABF alone.

Based on the observation that AANCP significantly inhibits Tau aggregation, we hypothesized that the inhibitory effect would be mediated by an enhanced clearance mechanism for pathological Tau. To confirm the hypothesized mechanism, the expression of key autophagy markers LC3-I/II, Beclin-1, and p62 was assessed in SH-SY5Y-TauP301L cells (Figure 6a). Treatment with ABF 10 μg/mL significantly upregulated LC3-II and Beclin-1 expression compared to the control group, suggesting an effect more pronounced than the results observed with LAA 80 μg/mL (Figure 6b,c). Correspondingly, ABF also produced a greater reduction in p62 expression, a protein degraded during autophagy, than LAA (Figure 6d). Crucially, AANCP (1:8 ratio) induced more substantial and favorable changes across all autophagy markers than ABF monotherapy. These findings suggest that the anti-aggregation activity of ABF and LAA is mediated through the promotion of autophagy and that AANCP possesses superior efficacy in autophagic clearance compared to each individual component.

## 3. Discussion

The development of effective therapies for AD is constrained by the complex and interconnected nature of Tau pathology, necessitating multifaceted therapeutic strategies [28]. The present study demonstrates that a novel AANCP offers a multipronged therapeutic strategy, effectively mitigating key pathological events in cellular models of AD. Our findings provide a strong proof-of-concept for AANCP as a multifaceted agent that not only prevents the initial dysregulation of Tau phosphorylation but also neutralizes the subsequent downstream toxic consequences.

The primary mechanism by which AANCP appears to exert protective effects on Tau pathology is through the robust regulation of Tau phosphorylation. We demonstrated that AANCP treatment leads to the inhibitory phosphorylation of GSK-3β at Ser9, which is a direct consequence of upstream Akt activation, a cornerstone of cell survival signaling [29]. This molecular response is consistent with previous reports on other natural polyphenols, such as cyanidin-3-glucoside, which modulate the Akt-GSK-3β signaling axis to reduce Tau pathology [30]. Simultaneously, AANCP enhanced the methylation of PP2A, boosting the activity of the major Tau phosphatase, a crucial mechanism for maintaining Tau in a dephosphorylated state [31,32]. By simultaneously suppressing a key Tau kinase and activating a major phosphatase, AANCP provides a powerful and efficient means to restore cellular homeostasis and prevent the accumulation of hyperphosphorylated Tau.

Furthermore, the results revealed that AANCP treatment significantly reduces t-Tau protein levels, rather than decreasing phosphorylated forms, involving enhanced protein clearance. This approach is supported by previous studies suggesting that pharmacological activation of autophagy is an effective strategy for clearing pathological Tau species from neuronal cells [33,34].

Beyond regulating phosphorylation and protein levels, AANCP directly dismantles the downstream progression of aberrant Tau. Our results reveal a two-pronged attack on pathological Tau aggregates: direct inhibition and enhanced clearance. The ThT fluorescence assay for detecting amyloid fibril formation [35] confirmed that AANCP physically disrupts the fibrillization process, with the 1:8 ratio showing particularly potent anti-aggregation activity. Concurrently, AANCP treatment stimulated autophagic machinery, evidenced by increased LC3-II and Beclin-1 levels and the degradation of the autophagy substrate p62 [36]. These results suggest that AANCP blocks the formation of new aggregates and promotes the elimination of existing toxic species through the cellular clearance system.

A critical finding of this study is the consistent synergistic efficacy of the two components constituting the AANCP formulation. While ABF was the principal active component, combination with LAA into a stable nanocomplex consistently yielded superior therapeutic effects compared to ABF alone. This synergy is attributable to the physicochemical advantages conferred by the nanocomplex structure, such as enhanced structural stability and improved cellular bioavailability of the active anthocyanins [27]. Intriguingly, the optimal synergistic ratios differed depending on the specific pathological target (1:1 for phosphorylation vs. 1:8 for aggregation). This finding suggests that the physicochemical properties of the nanocomplex are critical, as a 1:1 ratio appears ideal for modulating cell signaling receptors and kinases, whereas a 1:8 ratio is better suited for providing steric hindrance to block the physical docking and the fibrillization of Tau peptides.

While these findings are promising, the present study includes certain limitations. The experiments were conducted in transfected cell lines (HEK293 and SH-SY5Y), which may not fully recapitulate the complex pathophysiology of the human AD brain [37]. Therefore, future investigations are essential to validate these effects in biomimetic models, such as patient-derived iPSC neurons or brain organoids, which are becoming indispensable tools in neurodegenerative disease research [38]. Furthermore, evaluating the ability of AANCP to cross the blood-brain barrier [39] and performing comprehensive pharmacokinetic and pharmacodynamic studies will be crucial steps for clinical translation [40].

**Figure 7:**
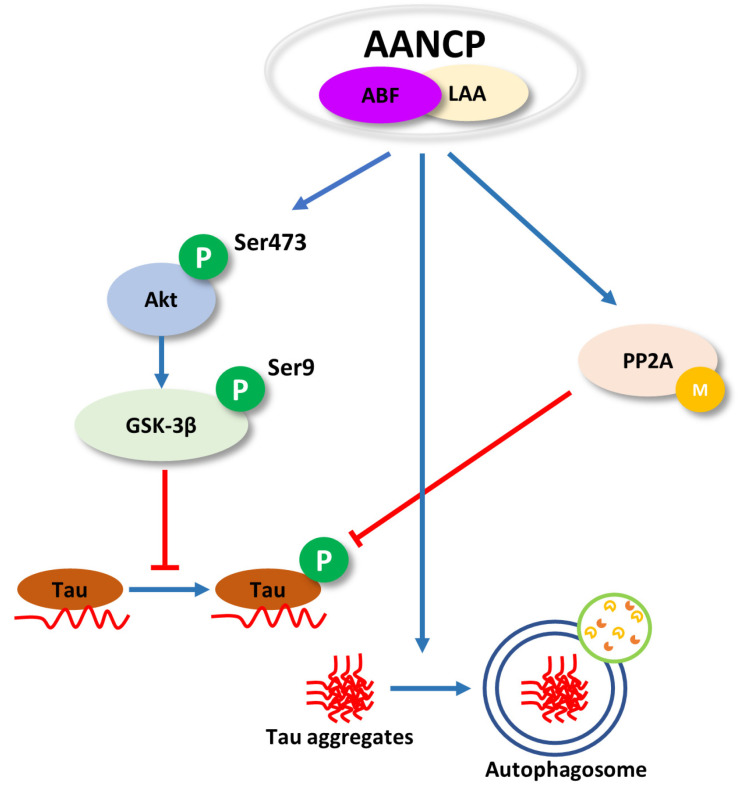
Schematic overview of the molecular mechanisms underlying the therapeutic effects of AANCP on Tau pathology. The schematic illustrates how AANCP combats key features of neurodegeneration in models of Alzheimer’s disease through three interconnected actions. (1) Regulation of Tau phosphorylation: AANCP activates the Akt survival pathway, leading to GSK-3β inhibition, and simultaneously boosts the activity of the phosphatase PP2A. (2) Inhibition of Tau aggregation: AANCP directly interferes with the fibrillization process of Tau peptides. (3) Enhancement of autophagic clearance: AANCP promotes the degradation of toxic Tau aggregates by increasing the expression of key autophagy markers LC3-II and Beclin-1, while reducing p62 levels. These effects are synergistically enhanced by the nanocomplex formulation.

In conclusion, by simultaneously targeting Tau pathology, including Tau phosphorylation, aggregation, and clearance, with a synergistic nanocomplex formulation, this study establishes AANCP as a promising therapeutic candidate for further development in the treatment of AD and other tauopathies (Figure 7).

## 4. Materials and Methods

### 4.1. Cell Culture

Human embryonic kidney (HEK293) cells were obtained from the Korean Cell Line Bank (Seoul, Republic of Korea), and SH-SY5Y (human neuroblastoma) cells were acquired from the American Type Culture Collection (Manassas, VA, USA). Both cell lines were maintained in Dulbecco’s Modified Eagle Medium (Welgene, Gyeongsan, Republic of Korea) supplemented with 10% fetal bovine serum (Gibco, Grand Island, NY, USA) and 1% antibiotic-antimycotic solution (Gibco, Grand Island, NY, USA). All cells were cultured in a humidified incubator at 37 °C with a 5% CO_2_ atmosphere.

### 4.2. Preparation of Compounds and Cellular Treatments

ABF and LAA were sourced from the JBK lab (Seongnam, Republic of Korea). Stock solutions were prepared in complete medium. To prepare the AANCP formulation, ABF and LAA were mixed at their designated ratios and incubated for 30 min at room temperature to allow for self-assembly. All solutions were diluted to their final working concentrations immediately before use.

For experiments, cells were seeded into 12-well plates at a density of 5 × 10^5^ cells/mL. After 24 h of stabilization, cells were assigned to various treatment groups. The “Mock” group consisted of cells that did not undergo transfection. The “Control” group consisted of transfected cells treated only with the vehicle solution. The “Positive control” group was treated with 10 mM LiCl as a well-known GSK-3β inhibitor (Sigma-Aldrich, St. Louis, MO, USA) and 1 μM TRx0237 mesylate (Selleckchem, Houston, TX, USA). Experimental groups were treated with ABF (1.0, 2.5, 5.0, or 7.5 μg/mL), LAA (1.0, 2.5, 5.0, or 7.5 μg/mL), or AANCPs, which consisted of a fixed concentration of ABF (2.5 μg/mL) combined with varying concentrations of LAA (1.0, 2.5, 5.0, or 7.5 μg/mL). Following treatment, the cells were incubated for an additional 24 h before being harvested for subsequent analyses.

### 4.3. Transient Transfection for Tau Overexpression

To induce Tau phosphorylation, HEK293 and SH-SY5Y cells were transiently transfected with a plasmid encoding the longest human Tau isoform with the P301L mutation, which was kindly provided by Professor Seon-Yong Jeong (Department of Medical Genetics, Ajou University, Suwon, Republic of Korea), using polyethyleneimine (~25 kDa PEI; Sigma-Aldrich, St Louis, MO, USA). Cells were seeded at 5 × 10^5^ cells/mL in 12-well plates and incubated for 24 h. The transfection complex was prepared by diluting the plasmid DNA and PEI separately in Opti-MEM (Gibco, Grand Island, NY, USA). The PEI solution was incubated for 10 min at room temperature before being combined with the DNA solution, using a PEI-to-DNA ratio optimized for maximal efficiency and minimal cytotoxicity. The resulting complex was then added dropwise to the cells, followed by a 24 h incubation period.

### 4.4. Protein Extraction and Quantification

Total cellular proteins were extracted by lysing the cells in radioimmunoprecipitation assay (RIPA) buffer (BIOSESANG, Seongnam, Republic of Korea) supplemented with a protease inhibitor cocktail and sodium orthovanadate (Sigma-Aldrich, St Louis, MO, USA). The lysis was performed on ice for 15 min. The resulting lysates were centrifuged at 15,000 rpm for 15 min at 4 °C to pellet insoluble debris. The protein concentration in the supernatant was determined using the DC Protein Assay kit (Bio-Rad, Hercules, CA, USA), with absorbance measured on a TriStar2 LB 942 Multimode Microplate Reader (Berthold Technologies, Bad Wildbad, Germany).

### 4.5. Western Blot Analysis

Equal amounts of protein (30 µg) from each sample were separated by 6–10% sodium dodecyl sulfate-polyacrylamide gel electrophoresis and subsequently transferred to polyvinylidene difluoride membranes (Merck Millipore, Burlington, MA, USA). The membranes were blocked for 1 h at room temperature with 5% skim milk or bovine serum albumin in Tris-buffered saline with Tween-20. Membranes were then incubated overnight at 4 °C with the following primary antibodies: Tau (#46687S), p-Tau (S396) (#9632S), GSK-3β (#9315S), p-GSK-3β (S9) (#9323S), Akt (#9272S), p-Akt (S473) (#9271S), LC3 (#12741T), Beclin-1 (#3495T), SQSTM1/p62 (#5114T), PP2AC (#2038S; Cell Signaling Technology, Danvers, MA, USA), methyl-PP2AC (L309) (ab66597; Abcam, Cambridge, UK), and GAPDH (sc-32233; Santa Cruz Biotechnology, Dallas, TX, USA). After washing, the membranes were incubated for 1 h at room temperature with the appropriate secondary antibody: horseradish peroxidase-conjugated goat anti-mouse IgG (A90-116P) or goat anti-rabbit IgG (A120-101P; both Bethyl Laboratories, Montgomery, TX, USA). Protein bands were visualized using West-Q Pico Dura ECL Solution (GenDEPOT, Katy, TX, USA) on an iBright CL1500 Imaging System (Thermo Fisher Scientific, Waltham, MA, USA). Densitometric analysis was performed using ImageJ software (version 1.54; NIH, Bethesda, MD, USA), with all target protein levels normalized to GAPDH levels.

### 4.6. Tau Aggregation Inhibition Assay

The inhibitory effects of the compounds on Tau aggregation were measured using a ThT fluorescence assay. A custom-synthesized peptide corresponding to TauP301L (Ac-DNIKHVLGGGSVQIVYK-NH_2_) was obtained from BIOSTEM (Suwon, Republic of Korea). The peptide was diluted to a final concentration of 50 or 100 µM in an aggregation buffer (20 mM Tris, pH 7.4, 100 mM NaCl, 1 mM EDTA). The experiment was set up in black 96-well plates with the following groups: a control group (peptide with heparin only), a positive control (10 µM TRx0237), and experimental groups treated with ABF (2, 5, or 10 μg/mL), LAA (20, 40, or 80 μg/mL), or AANCP (10 μg/mL ABF combined with 20, 40, or 80 μg/mL LAA). Tau aggregation was initiated by adding 300 μM heparin (Sigma-Aldrich). The plates were then incubated at 37 °C for 24 h with shaking. Following incubation, a ThT solution (3 mM ThT, 100 mM dithiothreitol; Sigma-Aldrich, St Louis, MO, USA) was added to each well. Fluorescence was measured on a FlexStation 3 Multi-Mode Microplate Reader (Molecular Devices, San Jose, CA, USA) with excitation at 440 nm and emission at 484 nm.

### 4.7. Statistical Analysis

All experiments were performed at least three times independently. Data are presented as the mean ± standard deviation. Statistically significant differences between two groups were assessed using an unpaired two-tailed Student’s *t*-test. Significance among multiple groups was determined using one-way ANOVA followed by an appropriate post hoc test. All analyses were performed using SPSS software (version 20; IBM, Armonk, NY, USA), with a *p*-value of <0.05 considered significant.

## 5. Conclusions

The findings of this study establish AANCP as a novel, potent, multifaceted modulator of Tau pathology in cellular models of AD. AANCP effectively attenuates Tau hyperphosphorylation by concurrently inhibiting GSK-3β through the Akt pathway and activating the phosphatase PP2A. Furthermore, the nanocomplex directly inhibits Tau aggregation and enhances the clearance of pathological Tau by upregulating autophagic flux. The synergistic activity observed with the AANCP consistently surpassed that of its individual components, positioning it as a promising multitarget therapeutic candidate for the future development of novel treatments for AD and other tauopathies.

## Data Availability

The original contributions presented in this study are included in the article. Further inquiries can be directed to the corresponding authors.

## Abbreviations

The following abbreviations are used in this manuscript:

AANCP	* Aronia * bioactive fraction-alginic acid nanocomplex
ABF	* Aronia * bioactive fraction
AD	Alzheimer’s disease
Aβ	amyloid-β
GSK-3β	glycogen synthase kinase-3β
HRP	horseradish peroxidase
LAA	low molecular weight alginic acid
p-Tau	phosphorylated Tau
PEI	polyethyleneimine
PP2A	protein phosphatase 2A
t-Tau	total Tau
ThT	thioflavin T
